# Procyanidins alleviates morphine tolerance by inhibiting activation of NLRP3 inflammasome in microglia

**DOI:** 10.1186/s12974-016-0520-z

**Published:** 2016-03-01

**Authors:** Yang Cai, Hong Kong, Yin-Bing Pan, Lai Jiang, Xiu-Xiu Pan, Liang Hu, Yan-Ning Qian, Chun-Yi Jiang, Wen-Tao Liu

**Affiliations:** Jiangsu Key Laboratory of Neurodegeneration, Department of Pharmacology, Nanjing Medical University, 140 Han-Zhong Road, Nanjing, 210029 China; Department of Anesthesiology, The First Affiliated Hospital of Nanjing Medical University, Nanjing, 210029 China

**Keywords:** Morphine tolerance, Procyanidins, NLRP3 inflammasome, Interleukin-1β, Microglia

## Abstract

**Background:**

The development of antinociceptive tolerance following repetitive administration of opioid analgesics significantly hinders their clinical use. Evidence has accumulated indicating that microglia within the spinal cord plays a critical role in morphine tolerance. The inhibitor of microglia is effective to attenuate the tolerance; however, the mechanism is not fully understood. Our present study investigated the effects and possible mechanism of a natural product procyanidins in improving morphine tolerance via its specific inhibition on NOD-like receptor protein3 (NLRP3) inflammasome in microglia.

**Methods:**

CD-1 mice were used for tail-flick test to evaluate the degree of pain. The microglial cell line BV-2 was used to investigate the effects and the mechanism of procyanidins. Reactive oxygen species (ROS) produced from BV-2 cells was evaluated by flow cytometry. Cell signaling was measured by western blot assay and immunofluorescence assay.

**Results:**

Co-administration of procyanidins with morphine potentiated its antinociception effect and attenuated the development of acute and chronic morphine tolerance. Procyanidins also inhibited morphine-induced increase of interleukin-1β and activation of NOD-like receptor protein3 (NLRP3) inflammasome. Furthermore, procyanidins decreased the phosphorylation of p38 mitogen-activated protein kinase, inhibited the translocation of nuclear factor-κB (NF-κB), and suppressed the level of reactive oxygen species in microglia.

**Conclusions:**

Procyanidins suppresses morphine-induced activation of NLRP3 inflammasome and inflammatory responses in microglia, and thus resulting in significant attenuation of morphine antinociceptive tolerance.

**Electronic supplementary material:**

The online version of this article (doi:10.1186/s12974-016-0520-z) contains supplementary material, which is available to authorized users.

## Background

Morphine as the gold standard for treating pain is widely used in clinic. However, long-term use of morphine leads to analgesic tolerance, which greatly reduces the utilization of this drug. The mechanism of morphine tolerance is complicated involving many aspects, such as receptors, ion channels and neural networks [1–3].

For a long period, most investigations focused morphine tolerance on neurons. A lot of evidences suggest that morphine activates opioid receptors, resulting in an endocytosis of μ-opioid receptor (MOR) and upregulated expression of *N*-methyl-d-aspartic acid (NMDA) receptors in the cell surface [4–7]. The phosphorylated NMDA receptors exhibit an enhanced calcium conductance [8], leading to an upregulation of protein kinase C (PKC) [6, 9, 10], which potentiates neuronal excitability and finally lead to opiate tolerance.

However, compelling evidences reported recently have shown that morphine tolerance is also due to the activation of microglia [11–13]. Chronic morphine exposure induces persistent activation of spinal cord microglia [14, 15]. Studies demonstrate that morphine binds to Toll-like receptor 4 (TLR4), leading to the initiation of innate immune signaling cascade and the production of proinflammatory factors [16].

The microglia activated by morphine releases proinflammatory cytokines, including interleukin (IL)-1β, tumor necrosis factor (TNF)-α, and IL-6 [6]. These cytokines enhance the hyperactivity of dorsal horn neurons, which cause the central sensitization and reduction in morphine analgesic efficacy [17]. As the most important factor in inflammatory processes [18], IL-1β has a unique molecular mechanism for its maturation in an intracellular multiprotein complex named as inflammasome. In microglia, pro-IL-1β was processed by NOD-like receptor protein3 (NLRP3) inflammasome and then the mature IL-1β was secreted [19].

NLRP3 is a unique inflammasome whose assembly and activation involves a two-step process. Firstly, a priming signal stimulates TLR4 and then enhances NF-κB-driven transcription of NLRP3 [20]. Then a second signal promotes NLRP3 to form a protein complex with apoptosis-associated speck-like protein (ASC) containing a caspase recruitment domain [21–23].

Currently, there are three models of NLRP3 activation to illuminate this process. First, the generation of reactive oxygen species (ROS) is considered to be critical for activation of NLRP3 inflammasome. ROS induces the dissociation of thioredoxin and then activates NLRP3 [24]. Second, extracellular ATP binds to P2X7R and triggers K^+^ efflux, which is a sufficient signal for NLRP3 activation [25–27]. Then, a third model has been proposed that certain particulate activators can activate NLRP3 inflammasome by Cathepsin B released from destabilized lysosomal compartment [28].

The function of NLRP3 inflammasome is crucial for the regulation of neuroinflammation mediated by microglia [19]. Therefore, compounds that inhibit the activation of NLRP3 inflammasome may contribute to the improvement of morphine tolerance. Procyanidins is a potent and safe natural product usually extracted from grape seed. It includes a structurally diverse set of compounds, with potentially hundreds of individual compounds with anti-oxidant, anti-inflammatory, and anti-allergic activities [29–31]. Furthermore, procyanidins is available from a wide variety of dietary sources: grapes, nuts, cocoa, apples, peaches, berries, pears, cranberries, and peanuts. Recently, it was recognized as health protective agent [32, 33]. Epidemiological studies indicated that populations consuming procyanidins-rich foods had lower incidences of inflammatory diseases, including metabolic syndrome and atherosclerosis [34, 35]. There is a large number of scientific evidence manifesting the inhibitory effects of procyanidins on inflammation [36].

Herein, we demonstrate for the first time that morphine can activate microglial NLRP3 inflammasome. Moreover, procyanidins, a safe and effective natural product, can attenuate morphine tolerance by inhibiting the NLRP3 activation of inflammasome and the maturation of IL-1β through the unique and potent efficacy of ROS clearance.

## Methods

### Ethics statement

All procedures were strictly performed in accordance with the regulations of the ethics committee of the International Association for the Study of Pain and the Guide for the Care and Use of Laboratory Animals (The Ministry of Science and Technology of China, 2006). All animal experiments were approved by the Nanjing Medical University Animal Care and Use Committee, and were designed to minimize suffering and the number of animals used.

### Animals

Adult CD-1 mice (18–22 g) were provided by the Experimental Animal Center at Nanjing Medical University, Nanjing, China. Mice were housed five to six per cage under pathogen-free conditions with soft bedding under controlled temperature (22 ± 2 °C) and a 12 h light/dark cycle (lights on at 8:00 a.m.). Behavioral testing was performed during the light cycle (between 9:00 a.m. and 5:00 p.m.). Mice were allowed to acclimate to these conditions for at least 2 days before inclusion in experiments. For each group of experiments, the animals were matched by age and body weight.

### Reagents

Procyanidins was purchased from Zelang Pharmaceutical Co. Ltd (Nanjing, China). The purity of procyanidins was more than 95 %. Procyanidins contained 1.1 % monomeric, 34.2 % dimeric, 24.9 % trimeric, 6.7 % tetrameric (totally 66.9 % oligomeric procyanidins), and 33.1 % polymeric procyanidins. IL-1β was from Santa Cruz Biotechnology (Santa Cruz, CA, USA). Antibodies for caspase-1 and NLRP3 were acquired from Adipogen International (San Diego, CA, USA). Antibody for glyceraldehyde-3-phosphate dehydrogenase (GAPDH) was from Sigma-Aldrich (St. Louis, MO, USA). Antibody for ionized calcium-binding adapter molecule 1 (IBA-1) was from Wako Pure Chemical Industries (Osaka, Japan). The antibodies for p38, c-Jun N-terminal kinase (JNK), extracellular-regulated protein kinases (ERK), NMDA-NR1, PKC, p65/RelA, and immunofluorescent antibody for IBA-1 were from Abcam (Cambridge, MA, USA). Antibodies for phosphorylated NR1 subunit (Ser896), phosphorylated PKC (pan) (gamma Thr514), phosphorylated p38 (Tyr182), phosphorylated ERK (Thr202/Tyr204), phosphorylated JNK (Thr183/Tyr185) and immunofluorescent antibody for c-fos were from Cell Signaling Technology (Beverly, MA, USA). Immunofluorescent antibody for calcitonin gene related peptide (CGRP) was from Santa Cruz Biotechnology (Santa Cruz, CA, USA). Lipopolysaccharide (LPS), rotenone, dimethyl sulfoxide (DMSO), and ATP were purchased from Sigma-Aldrich (St. Louis, MO, USA). Morphine hydrochloride was purchased from Shenyang First Pharmaceutical Factory, Northeast Pharmaceutical Group Company (Shenyang, China). Fetal bovine serum (FBS) was purchased from Gibco, and other cell culture media and supplements were purchased from HyClone (Logan, UT, USA). 3-(4,5-dimethyl-2-thiazolyl)-2,5-diphenyl-2-H-tetrazolium bromide (MTT) was purchased from Sunshine Biotechnology (Nanjing, China). MitoSOX was purchased from Thermo Fisher Scientific (Waltham, MA, USA). All other reagents were from Sigma-Aldrich (St. Louis, MO, USA).

### Cell preparation and stimulation

BV-2 cells were maintained in humidified 5 % CO2 at 37 °C in Dulbecco’s modified Eagle’s Medium (DMEM) supplemented with 10 % (*v*/*v*) FBS, penicillin (100 U/ml), and streptomycin (100 U/ml). For inducing inflammasome activation, 10^5^ cells were plated in 6-well plate overnight and the medium were changed to serum-free medium in the following morning and then the cells were treated with morphine (200 μM) or LPS (1 μg/ml) with or without procyanidins (1 ‰ DMSO) for 12 h. We performed the DMSO-only as the control. After that, the BV-2 cells were stimulated with ATP (5 mM) for 1 h. Cell extracts and precipitated supernatants were analyzed by immunoblotting.

### Tolerance models and behavioral analysis

We habituated animals in the testing environments for 2 days and carried out behavioral testing in a blinded manner. For the test of chronic tolerance, mice were injected with saline or morphine (10 mg/kg) subcutaneously every 12 h for 7 days and analgesia was assessed 0.5 h after morphine administration by the tail-flick assay every morning [15]. Briefly, mice’s tails were placed in 55 °C hot water, and the latency of tail withdrawal was measured. A cut-off time of 30 s was set to avoid tissue damage. Procyanidins was suspended in 0.5 % sodium carboxymethyl cellulose (CMC-Na). Procyanidins (20, 40, or 80 mg/kg) was administered intragastrically 15 min before morphine given twice a day from day 1 to day 7.

### Measurement of cytokines

Mouse IL-1β in supernatants from cell culture were determined using the ELISA kits (R&D Systems, Minneapolis, MN, USA) according to manufacturer’s instructions. Level of malondialdehyde (MDA) was assayed with kits from Nanjing Jiancheng Bioengineering Institute (Nanjing, China) according to manufacturer’s instructions.

### NF-κB activation assay

BV-2 cells were plated in class bottom cell culture dishes and treated with morphine (200 μM) for 4 h with or without procyanidins (100 μM). Then BV-2 cells were fixed with ice-cold methanol and were permeabilized with 0.25 % Triton X-100/PBST. After blocking with 1 % bovine serum albumin (BSA) in PBST for 1 h, the coverslips with BV-2 cells were incubated for 2 h at room temperature with the p65/RelA antibody diluted in 1 % BSA (1:50). Then the coverslips were exposed to the fluorescein isothiocyanate (FITC)-conjugated anti-rabbit IgG (1:100, at room temperature for 1 h) and then were rinsed three times with PBS. Finally, the coverslips were stained with 1 μg/mL DAPI (4′,6-diamidino-2-phenylindole, a fluorescent DNA dye to mark nucleus) for 1 min. Confocal microscopy analyze was carried out using Olympus FV1000 confocal system.

### ROS measurement

BV-2 cells were plated in non-tissue-culture-treated six-well dishes and stimulated with morphine (200 μM) for 12 h with or without the pre-treatment of procyanidins (100 μM). A positive control was stimulated with rotenone (10 μM) for 6 h. After the cultivation, supernatant was removed and cells were washed with PBS. Then the cells were incubated with MitoSOX (to measure mitochondria-associated ROS levels) at 2.5 μM in serum-free DMEM for 0.5 h at 37 °C. After that, cells were washed with warm PBS, removed from plates with cold PBS containing 1 % FBS, and subjected to fluorescence-activated cell sorting (FACS) analysis (Miltenyi MACSQuant Analyzer 10, Germany). The data were analyzed using FlowJo statistical software (Emerald Biotech Co., Ltd).

### Western blot

Samples (cells or spinal cord tissue segments at L1-L6) were collected and washed with ice-cold PBS before being lysed in radio immunoprecipitation assay (RIPA) lysis buffer [Beyotime, Shanghai, China; 50 mmol/L Tris (pH 7.4), 150 mmol/L NaCl, 1 % Triton X-100, 1 % sodium deoxycholate, 0.1 % sodium dodecyl sulfate, 1 mmol/L phenylmethylsulfonyl fluoride, 0.15 U/mL aprotinin, and 1 mg/mL pepstatin] and then sample lysates were separated by SDS-PAGE and electrophoretically transferred onto polyvinylidene fluoride membranes (Millipore Corp., Bedford, MA, USA). The membranes were blocked with 10 % whole milk in TBST (Tris–HCl, NaCl, Tween 20) for 2 h at room temperature, probed with primary antibodies at 4 °C overnight [GAPDH, 1:8000; IBA-1, 1:1000; NLRP3, 1;1000; caspase-1, 1:1000; IL-1β, 1:500; TNF-α, 1:1000; p-p38 (Tyr182), 1:1000; p38, 1:1000; p-ERK, 1:1000; ERK, 1:1000; p-JNK, 1:1000; JNK, 1:1000; p-NR1(Ser896), 1:1000; NR1, 1:1000; p-PKC, 1:1000; PKC, 1:1000;] and then incubated with horseradish peroxidase-coupled secondary antibodies from Cell Signaling Technology (Beverly, MA, USA). Data were acquired with the Molecular Imager (Gel DocTM XR, 170–8170) and analyzed with Quantity One-4.6.5 (Bio-Rad Laboratories, Berkeley, CA, USA).

### Immunofluorescence assay

Under deep anesthesia by intraperitoneal injection of sodium pentobarbital (100 mg/kg), the animal was perfused with normal saline followed by 4 % paraformaldehyde in 0.1 M PBS, pH 7.2–7.4, for 20 min. Then L4 and/or L5 lumbar segment were dissected out and post-fixed in the same fixative. The embedded blocks were sectioned as 30-μm thick and processed for immunofluorescence assay. Sections from each group (five mice in each group) were incubated with primary antibody (IBA-1, 1:200; c-fos, 1:200; CGRP, 1:200; NeuN, 1:300). Then the free-floating sections were washed with PBS, and incubated with the secondary antibody (1:300; Jackson Laboratories, USA) for 2 h at room temperature. After being washed three times with PBS, the samples were investigated with a confocal microscope (Leica TCS SPEII, Leica Biosystems, Wetzlar, Germany) for morphologic details. Images were randomly coded and transferred to a computer for further analysis.

### Statistical analysis

SPSS Rel 15 (SPSS Inc., Chicago, IL, USA) was used to conduct all the statistical analyses. Data were statistically evaluated by two-way analysis of variance (ANOVA) followed by Bonferroni post hoc tests. The mean fluorescent pixels of IBA-1 and CGRP were measured by Image Pro Plus 6.0 (Media Cybernetics, Silver Spring, MD, USA). Results were represented as mean ± standard error of the three independent experiments. Results described as significant were based on a criterion of *p* < 0.05.

## Results

### Systemic administration of procyanidins improved the morphine tolerance and suppressed morphine-induced microglial activation in the spinal cord

**Fig. 1:**
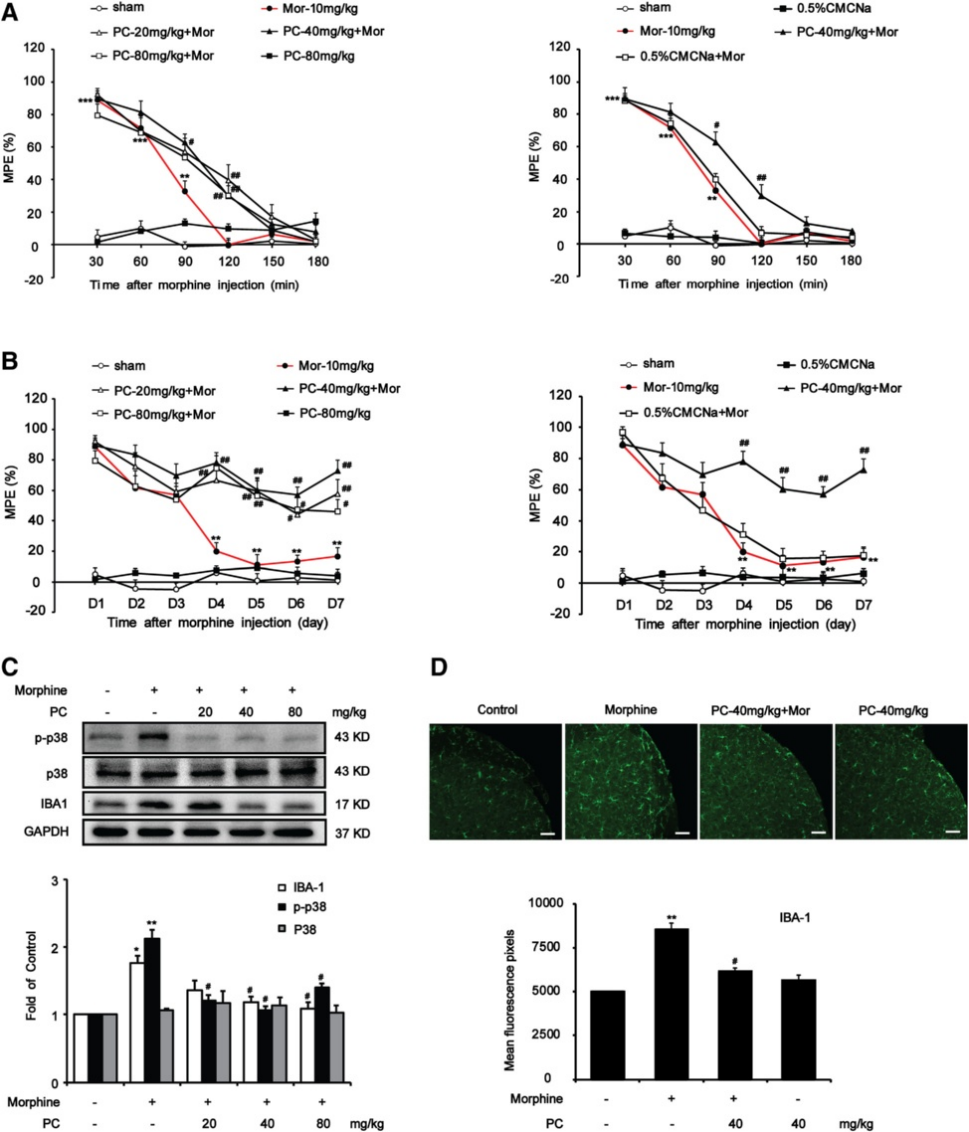
Procyanidins suppressed acute and chronic morphine tolerance via inhibiting microglia in the spinal cord. Tail-flick method was performed to evaluate the effect of procyanidins on the morphine tolerance. Data (*n* = 8) were shown as percentage of maximal possible effect (% MPE). **a** Procyanidins suppressed acute morphine tolerance. Before the treatment of morphine (10 mg/kg, s.c.), mice were pre-treated with different doses of procyanidins (i.g.) for 15 min. MPE was measured after morphine 10 mg/kg (s.c.) administration at the first day. **b** Procyanidins suppressed chronic morphine tolerance. Morphine was injected subcutaneously twice a day, and the MPE was measured 0.5 h after the first injection of each day. Before the treatment of morphine (10 mg/kg, s.c.), mice were pre-treated with different doses of procyanidins (i.g.) for 15 min. **c** Procyanidins inhibited morphine-induced upregulation of IBA-1 and phosphorylation of p38 MAPK, but not the p38 total protein. Samples were obtained and analyzed 2 h after morphine treatment on day 7. Representative western blot data (*n* = 4) for p-p38, p38, and IBA-1 was shown. **d** Immunofluorescence images and analysis showed the activation of microglia after morphine injection (*n* = 4) in the spinal cord. *p < 0.05, **p < 0.01, ***p < 0.001 vs. baseline; ^#^ *p* < 0.05, ^##^ *p* < 0.01 vs. morphine-treated group. Scale bar 75 μm

Some experiments were performed to investigate the role of procyanidins on morphine-induced tolerance in vivo. Procyanidins did not alter the pain threshold of mice up to 80 mg/kg. Data suggested that procyanidins significantly improved acute and chronic morphine tolerance (Fig. 1a, b). The MPE decreased to 16.52 % in chronically morphine-treated mice on day 7. The reduction in morphine’s MPE was significantly prevented by twice-daily coadministration of procyanidins (20, 40, or 80 mg/kg, i.g.). MPE decreased to 57.76, 72.88, and 46.11 %, respectively (Fig. 1b).

Subcutaneous injection of morphine induced activation of microglia in the spinal cord. Western blot and immunofluorescence data showed that repeated morphine treatment (10 mg/kg, twice a day for 7 days) resulted in an upregulation of the IBA-1 (a microglial marker) (Fig. 1c, d). Systemic administration of procyanidins inhibited morphine-induced microglial activation. Strikingly, 40 mg/kg procyanidins nearly reversed the increase of IBA-1 (Fig. 1c, d). Procyanidins also significantly reduced the upregulation of p38 MAPK phosphorylation induced by morphine exposure in the spinal cord (Fig. 1c). These data provided additional evidences that procyanidins could inhibit morphine-induced microglia activation.

### Procyanidins inhibited morphine-induced NLRP3 inflammasome activation in vivo

**Fig. 2:**
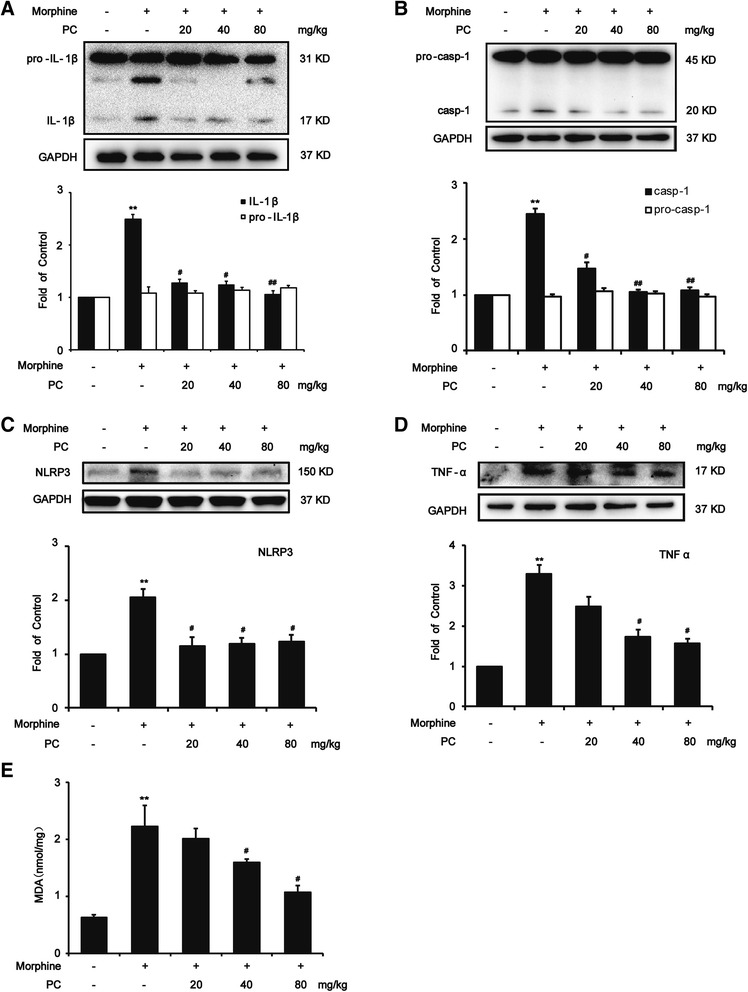
Procyanidins suppressed morphine-induced NLRP3 inflammasome activation in the spinal cord. **a** Procyanidins inhibited the morphine-induced upregulation of IL-1β in the spinal cord. **b** Procyanidins suppressed the morphine-induced activation of caspase-1 in the spinal cord. **c** Procyanidins inhibited the morphine-induced upregulation of NLRP3 in the spinal cord. **d** Procyanidins inhibited the morphine-induced upregulation of TNF-α in the spinal cord. The western blot samples (*n* = 4) were collected as described in methods. **e** Procyanidins inhibited the morphine-induced upregulation of malondialdehyde (MDA) in the spinal cord (*n* = 4). *p < 0.05, **p < 0.01 vs. baseline; ^#^ *p* < 0.05, ^##^ *p* < 0.01 vs. morphine-treated group

It was reported that morphine tolerance led to increased inflammatory response, especially the upregulation of IL-1β [6, 37]. Our data showed that consistent morphine exposure (10 mg/kg, twice a day for 7 days) increased protein levels of the proinflammatory cytokines IL-1β and TNF-α in mouse spinal cords. The repetitive administration of procyanidins (i.g., 15 min before each injection of morphine daily) suppressed the upregulation of proinflammatory cytokines in an efficient manner (Fig. 2a, d). IL-1β was processed by NLRP3 inflammasome in microglia [38]. Our data suggested that morphine exposure activated NLRP3, and procyanidins could inhibit this activation (Fig. 2c). Western blot analysis revealed that procyanidins could significantly reduce the increased protein levels of the caspase-1 (Fig. 2b) and the NLRP3, which played a well-established role in neuroinflammation.

MDA level can indirectly reflect attack severity of the body by ROS. Treatment of mice with morphine (10 mg/kg, twice a day for 7 days) caused an increase of the MDA level in the spinal cord, while pre-treatment with procyanidins markedly attenuated the increasing level of MDA (Fig. 2e).

### Procyanidins attenuated chronic morphine tolerance by inhibiting the phosphorylation of NMDA-NR1, PKC, and MAPKs in the spinal cord

**Fig. 3:**
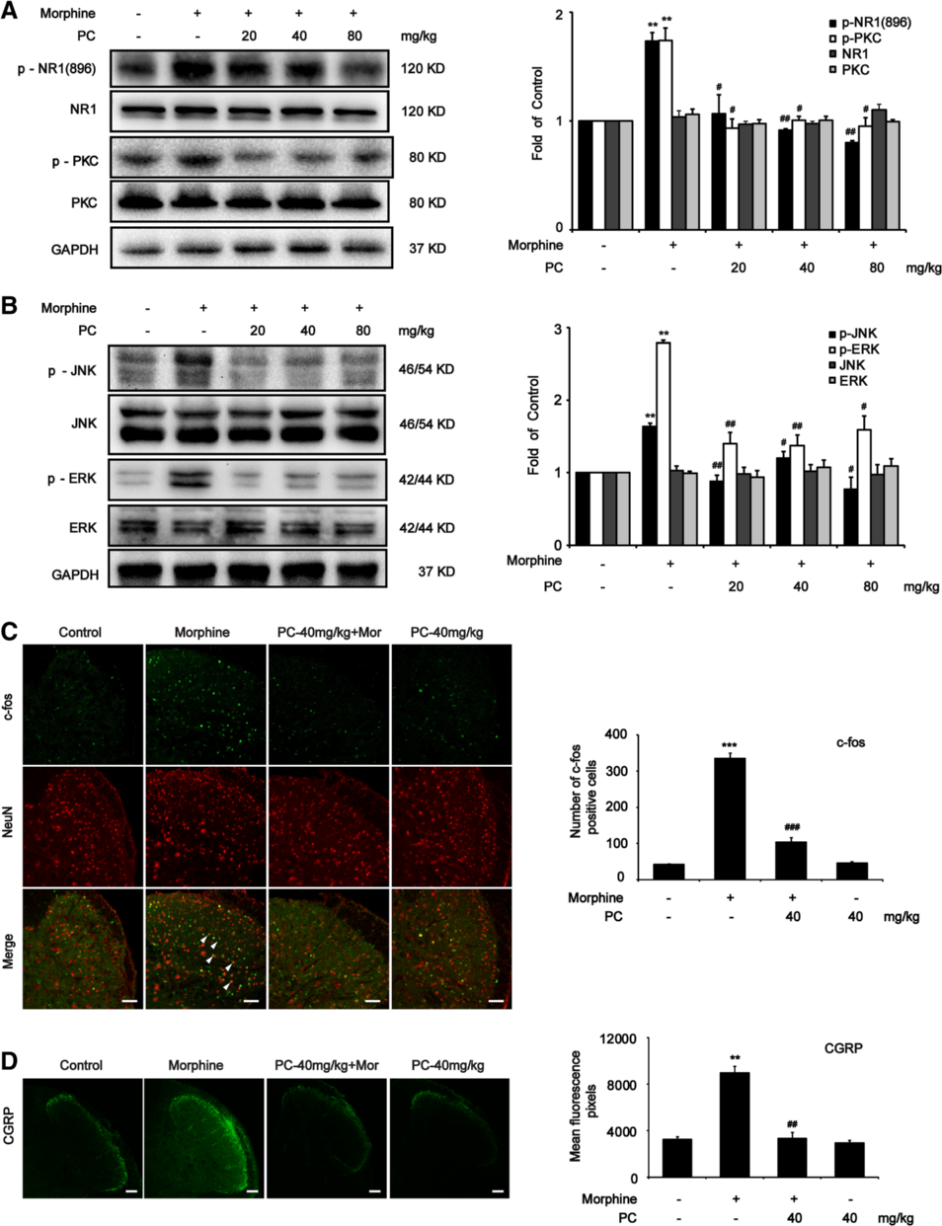
Procyanidins attenuated chronic morphine tolerance by inhibiting the neuron-glia pathway in the spinal cord. **a** Procyanidins inhibited the morphine-induced upregulation of phosphorylated PKC and phosphorylated NMDAR-NR1 in the spinal cord. The western blot samples (*n* = 4) were collected 2 h after the last morphine treatment. **b** Procyanidins inhibited the morphine-induced phosphorylation of JNK and ERK in the spinal cord. The western blot samples (*n* = 4) were collected as above. **c**, **d** Confocal images and immunofluorescence analysis showed the activation of neuronal c-fos and CGRP after morphine treatment (*n* = 4) in the spinal cord. The quantification of c-fos and CGRP immunofluorescence was respectively represented as number of c-fos positive cells and mean fluorescence pixels in the superficial dorsal horns. *p < 0.05, **p < 0.01, ***p < 0.001 vs. baseline; ^#^ *p* < 0.05, ^##^ *p* < 0.01, ^###^ *p* < 0.001 vs. morphine-treated group. Scale bar 75 μm

Studies show that activated microglia released proinflammatory cytokines, such as IL-1β and TNF-α, which stimulated neuron and glia through IL-1 receptor, NMDA receptor, and TNF receptor, resulting in the phosphorylation of PKC and MAPKs [37]. As a result, morphine tolerance and hyperalgesia were developed. We investigated the role of procyanidins on morphine-induced activation of neuron and glia in vivo. Western blot analysis revealed that procyanidins suppressed the morphine-induced upregulation of phosphorylated NMDAR-NR1 subunit and PKC in the spinal cords (Fig. 3a). Confocal images and immunofluorescence analysis showed that procyanidins could inhibit the activation of neuronal c-fos and CGRP after morphine treatment in the spinal cord (Fig. 3c, d). Furthermore, procyanidins also inhibit the increased phosphorylation of JNK and phosphorylation of ERK after morphine treatment (Fig. 3b).

### Procyanidins suppressed morphine-induced NLRP3 inflammasome activation in vitro

**Fig. 4:**
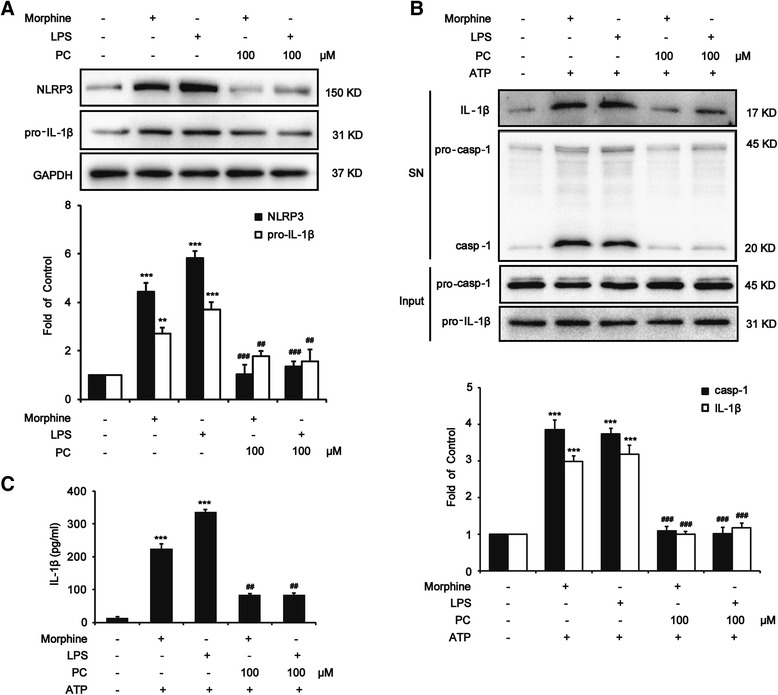
Procyanidins suppressed morphine-induced NLRP3 inflammasome activation in microglia. BV-2 cells were stimulated by LPS (1 μg/ml) or morphine (200 μM) with or without 100 μM of procyanidins for 12 h, and then, the inflammasome was activated with 5 mM of ATP for 1 h. **a** Procyanidins (100 μM) inhibited both LPS-induced and morphine-induced expression of pro-IL-1β and NLRP3 in BV-2 cells. The western blot samples for BV-2 cells (*n* = 4) were collected and analyzed 12 h after the last morphine exposure. **b** Procyanidins (100 μM) inhibited the morphine-induced and LPS-induced increase of caspase-1 and IL-1β in the supernatant of BV-2 cells. The western blot samples (*n* = 4) came from the supernatant of BV-2 cells. **c** ELISA of IL-1β in supernatants from LPS- and morphine-primed BV-2 cells and then stimulated with ATP. *p < 0.05, **p < 0.01, ***p < 0.001 vs. baseline; ^#^ *p* < 0.05, ^##^ *p* < 0.01, ^###^ *p* < 0.001 vs. morphine-treated group

To study the effects of procyanidins on morphine-induced microglial NLRP3 inflammasome activation in vitro, immortalized murine microglial cell line BV-2 was used [39, 40]. BV-2 cells were treated with morphine (200 μM) and different concentrations of procyanidins for 12 h with or without ATP for 1 h. Morphine treatment triggered the first step of activating NLRP3 inflammasome, which enhancing the level of NLRP3 and pro-IL-1β (Fig. 4a), and activated the second step, leading to the increasing level of IL-1β and caspase-1 in the supernatant (Fig. 4b, c). Compared with the only morphine-treated group, pre-administrated group with procyanidins (100 μM) 15 min before morphine administration significantly reduced the level of pro-IL-1β, IL-1β, NLRP3, and caspase-1 in BV-2 cells (Fig. 4). Moreover, procyanidins also suppressed the upregulation of NLRP3 inflammasome caused by classical TLR4 agonist LPS (Fig. 4) in microglia.

**Fig. 5:**
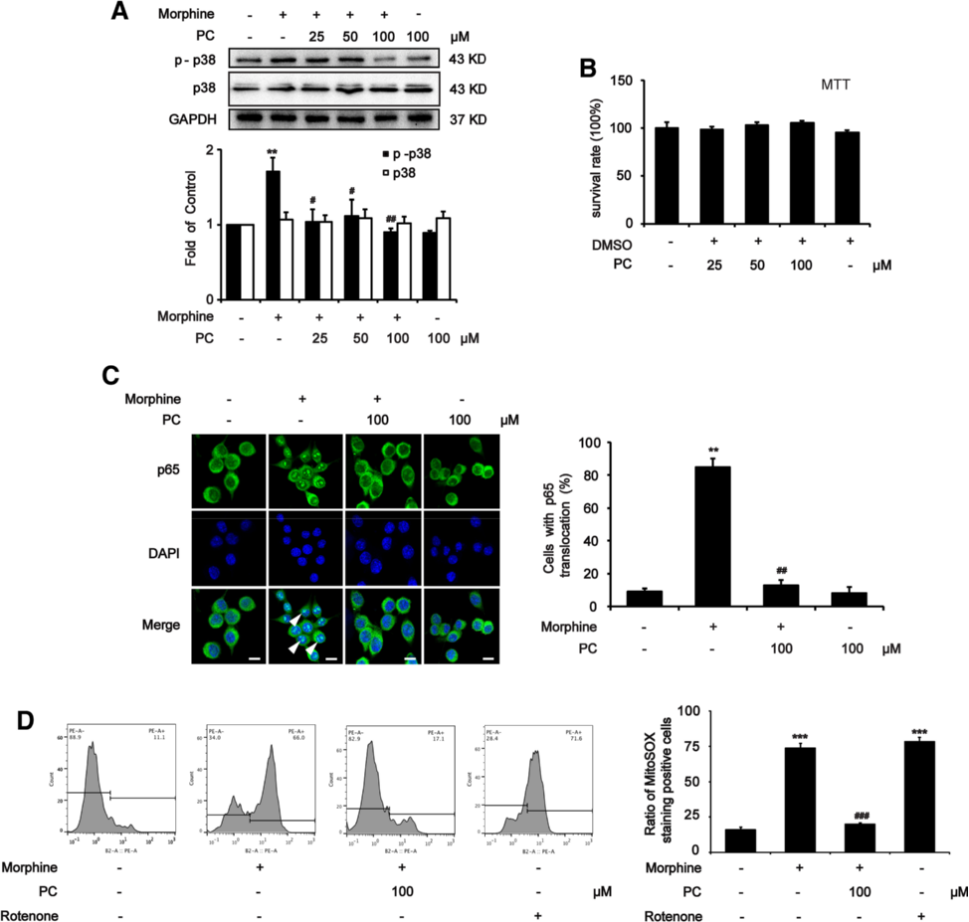
Procyanidins suppressed morphine tolerance by inhibiting p38 MAPK/NF-κB and ROS signaling in microglia. Before the treatment of morphine (200 μM), BV-2 cells were pre-treated with different doses of procyanidins. Then we handle and analyze BV-2 cells after morphine exposure. **a** Procyanidins inhibited the phosphorylation of p38 MAPK after morphine treatment for 2 h in a dose-dependent manner. Representative western blot bands and a data summary (*n* = 4) for p-p38 and p38 are shown. **b** MTT experiments showed that different doses of procyanidins did not affect cell proliferation. **c** Procyanidins (100 μM) inhibited the NF-κB translocation from the cytosol to the nucleus after morphine exposure for 4 h in BV-2 cells. **d** The level of ROS was assessed by calculating the ratio of MitoSOX staining positive cells to 10,000 cells through flow cytometry. Procyanidins (100 μM) inhibited the release of mitochondrial ROS after morphine treatment for 12 h in BV-2 cells. *p < 0.05, **p < 0.01, ***p < 0.001 vs. baseline; ^#^ *p* < 0.05, ^##^ *p* < 0.01, ^###^ *p* < 0.001 vs. morphine-treated group. Scale bar 20 μm

The p38 MAPK phosphorylation and p65 NF-κB translocation from the cytoplasm to the nucleus were shown to promote the maturation of NLRP3 inflammasome as the first activation signals in microglia [37]. Compared with the only morphine-treated group, pre-administrated (15 min) with procyanidins significantly reduced these effects (Fig. 5a, c). However, in BV-2 cells, procyanidins (100 μM) alone showed no significant effects on the phosphorylation of p38 MAPK and the translocation of p65 NF-κB (Fig. 5a, c). MTT assay indicated that the different doses of procyanidins did not affect cell proliferation (Fig. 5b).

Procyanidins as a potent free radical scavenger has demonstrated the ability to clear intracellular ROS. The main source of cellular ROS is mitochondria [41]. Various stresses including increased metabolic rates, hypoxia, or inflammatory response could markedly increase the production of mitochondrial ROS, which would activate NLRP3 inflammasome as the second activation signal in microglia [38]. To investigate a possible mechanism of morphine in inflammasome activation, we measured ROS production in mitochondria by flow cytometry [42]. Morphine significantly increased the level of ROS compared with the negative and positive control (Fig. 5d). Pre-administrated (15 min) with procyanidins (100 μM) significantly reduced morphine-induced production of ROS (Fig. 5d) and procyanidins also inhibited the ROS induced by rotenone (Additional file 1: Figure S1). 

## Discussion

In this study, we found procyanidins, a clinically used health product, had a significant inhibitory effect on morphine-induced activation of microglia by suppressing NLRP3 inflammasome activation. Therefore, procyanidins attenuated the development of acute and chronic morphine tolerance markedly. This study may provide a new solution to improve clinical analgesic efficacy of opioids through the inhibition of microglial NLRP3 inflammasome.

Procyanidins, as safe and effective compounds, can be absorbed from the gastrointestinal tract when people eat certain fruits and vegetables. It was usually administrated orally [43, 44]. However, the oral bioavailability of procyanidins was about 3–4 %, which was not very satisfactory though it had reached the standard of many clinically used drugs such as levodopa, a drug used to treat Parkinson’s disease [44–46]. More fortunately, procyanidins was a relatively safe drug with an oral LD_50_ value of over 4000 mg/kg. The optimal dose for the attenuation of morphine tolerance is 40 mg/kg in mice, which equals to 3.33 mg/kg in human. Therefore, human with a body weight of 60 kg might intake 400 mg of procyanidins every day if it is given twice a day. At present, a lot of commercial nutrition supplements containing procyanidins recommend a dosage from 400 to 800 mg every day. Therefore, procyanidins would be safe and effective for inhibiting morphine tolerance. These results indicate that it may be beneficial for the patients treated with opiate drugs to actively intake foods rich in procyanidins, such as: cranberry, blueberry, apple, cherry, and grape.

**Fig. 6:**
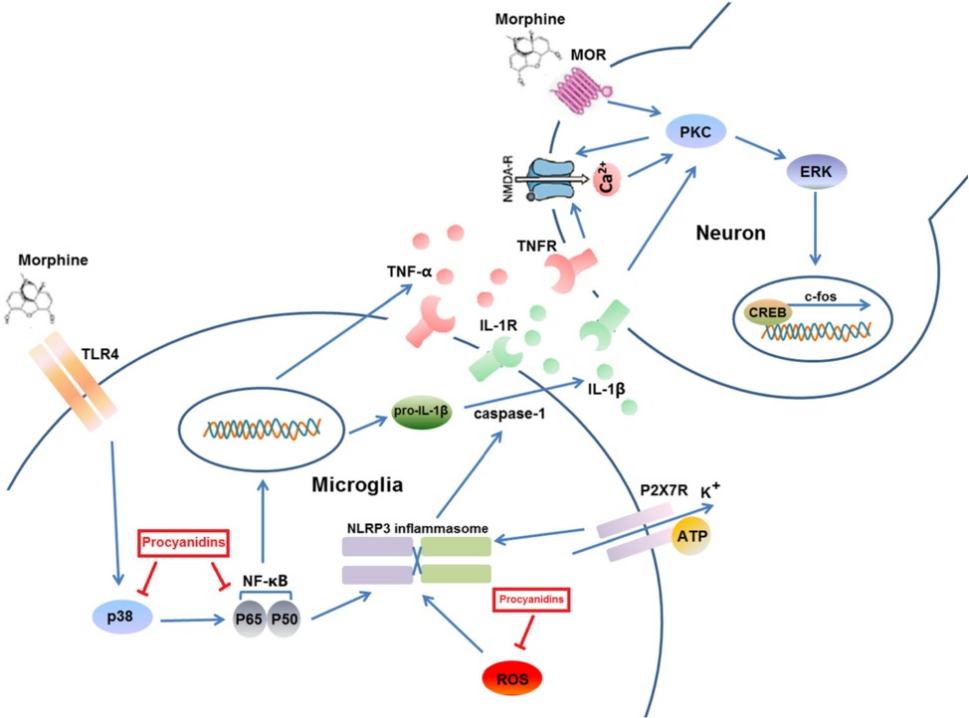
Schematic indicating the suppression of microglial NLRP3 to alleviate morphine tolerance by procyanidins. Upon the stimulation by morphine or LPS, several pathways including the release of ROS, and K^+^ efflux have been identified to signal the activation of NLRP3 inflammasome, leading to the activation of caspase-1 and the maturation of IL-1β. Procyanidins inhibited the ROS production and NLRP3 activation. The p38/NK-κB pathway was activated by morphine or LPS to induce the gene expression of NLRP3. The inhibition of p-p38 and NF-κB were a critical mechanism for the NLRP3 inflammasome suppressive effect of procyanidins in microglia

In the spinal cord, microglia regulated morphine tolerance via TLR4 signaling [47]. When morphine bound with TLR4, it activated the downstream intracellular signaling pathways similar to those activated by IL-1β binding to its cognate receptor, resulting in a potent proinflammatory signal (Fig. 6) [6, 48]. Our data showed that, pre-treatment of procyanidins significantly suppressed the level of IL-1β in vivo and in vitro after morphine exposure. Beside of this, adiministration of procyanidins alone did not affect the level of proinflammatory cytokines (Additional file: 1 Figure S2). In microglia, the NLRP3 inflammasome is a molecular platform activated upon signals of cellular “danger” to trigger innate immune defenses through the maturation of proinflammatory cytokines such as IL-1β [38]. We found that procyanidins could inhibit morphine-induced activation of NLRP3 inflammasome in vitro in the concentration of 100 μM. Though this concentration seems a little high, it could be achievable in vivo. Since the oral bioavailability of procyanidins was about 3–4 % [44–46], the blood concentration of 100 μM might be easily achieved when 80 mg/kg of procyanidins was given to mice which had a blood volume of 1.5 ml. However, further investigations are needed into the preclinical pharmacokinetic evaluation of procyanidins, especially on its distribution in the central nervous system.

Some studies reported that spinal P2X7R was involved in the induction but not maintenance of morphine tolerance [27]. As previously mentioned, the activation of P2X7R was viewed as the second signal for the activation of NLRP3 inflammasome in microglia (Fig. 6). It was reported that NAC, a ROS inhibitor, decreased IL-1β synthesis via inhibiting the P2X7R activation in murine macrophages [49]. Therefore, we could not rule out that procyanidins inactivated NLRP3 inflammasome by inhibiting P2X7R after morphine exposure in microglia.

Mao et al. reported that LPS-induced upregulation of NLRP3 was suppressed in the morphine-tolerant state, which may be one of the mechanisms involved in morphine-induced immunosuppression [50]. But to our best knowledge, we first confirmed that in the morphine-tolerant state, morphine could significantly activate NLRP3 inflammasome in the microglia.

It said before, ROS could induce the dissociation of thioredoxin and then activate NLRP3 inflammasome (Fig. 6). Our compound, procyanidins, as a ROS scavenger, significantly inhibited the morphine-induced release of ROS and then suppressed the microglial NLRP3 inflammasome to attenuate morphine tolerance (Fig. 6). Generation of IL-1β requires a priming signal and activating signal. The priming signal could be mediated by pattern recognition receptor (PRRs) such as TLRs, which would activate NF-κB (Fig. 6) [51]. The activated NF-κB can promote the transcription of NLRP3 (Fig. 6); therefore, we further investigated the effect of procyanidins on NF-κB activation. The mammalian NF-κB family members can be regulated by upstream diverse signal pathways, such as TLRs, TNF receptors, IL-1 receptors, and MAPKs [52, 53]. It was confirmed that morphine could activate TLR4, TNF-α, and IL-1β in microglia [54]. Furthermore, microglial p38 MAPK was dramatically and primarily activated by chronic morphine exposure in the spinal cord [27, 55, 56]. Indeed, our study revealed procyanidins could sufficiently inhibit morphine-induced NF-κB p65 nuclear translocation and remarkably reduced phosphorylation of p38 MAPK.

Morphine tolerance involves many receptors, channels, and processes. In a positive feedback loop, morphine induces activation of microglia releasing a lot of proinflammatory cytokines, which results in an extra-excitation of sensor neurons in the spinal cord [57–59] and decreases the efficiency of morphine analgesia [60]. This process involves the activation of NMDA/ERK/PKC signaling, which plays an important role in morphine tolerance [6]. The proinflammatory cytokines such as TNF-α and IL-1β activate NMDA receptor by phosphorylating PKC followed by a strong activation of ERK (Fig. 6). Then the phosphorylated ERK and PKC can facilitate many channels and receptors such as calcium channel on the cell membrane, resulting in the central sensitization and morphine tolerance (Fig. 6) [6, 61, 62]. In this study, we found that procyanidins notably inhibited the phosphorylation of NMDA, ERK, and PKC in the spinal cord. In addition to ERK and p38, we also found that procyanidins significantly inhibited the phosphorylation of JNK, which involved in the late stage of morphine tolerance [63, 64]. It indicated that procyanidins might also have significantly inhibitory effect in the late stage of morphine tolerance.

## Conclusions

In summary, procyanidins could extend acute morphine analgesia and attenuate chronic morphine tolerance with a possible biological mechanism of inhibiting neuroinflammation represented by microglia activation. Our results demonstrated that morphine exposure leading to excess production of ROS from mitochondria, and that procyanidins could diminish ROS and consequently inhibit the activation of NLRP3 inflammasome in microglia. Furthermore, procyanidins could suppress the over-activated NF-κB and MAPKs signaling, which played important roles in morphine tolerance. Altogether, our studies suggest that procyanidins may be a potential drug candidate to reduce morphine tolerance and to enhance the clinical utility of opioid drugs.

## Additional files

Additional file 1: Figures S1 and S2. BV2 cells were treated with rotenone for 6 h with or without the pre-treatment of procyanidins. We found that procyanidins could inhibit the upregulation of ROS which induced by rotenone. Additional controls with procyanidins treatment alone did not affect the baseline levels of NLRP3, pro-casp-1, casp-1, pro-IL-1β, IL-1β, and TNF-α. (DOCX 237 kb)

## Abbreviations

ASC: apoptosis-associated speck-like protein
BSA: bovine serum albumin
CGRP: c-fos and calcitonin gene-related peptide
CMC-Na: sodium carboxymethyl cellulose
DAPI: 4′,6-diamidino-2-phenylindole
DMEM: Dulbecco’s modified Eagle’s medium
DMSO: dimethyl sulfoxide
ERK: extracellular regulated protein kinases
FACS: fluorescence-activated cell sorting
FBS: fetal bovine serum
FITC: fluorescein isothiocyanate
GAPDH: glyceraldehyde-3-phosphate dehydrogenase
IBA-1: ionized calcium binding adapter molecule 1
IL-1β: interleukin-1β
IL-6: interleukin-6
JNK: c-Jun N-terminal kinase
LPS: lipopolysaccharide
MAPK: mitogen-activated protein kinase
MDA: malondialdehyde
MOR: μ-opioid receptor
MTT: 3-(4,5-dimethyl-2-thiazolyl)-2,5-diphenyl-2-H-tetrazolium bromide
NAC: *N*-acetyl-cysteine
NF-κB: nuclear factor-κB
NLRP: NOD-like receptor family pyrin domain-containing protein
NMDA: *N*-methyl-d-aspartic acid
PBS: phosphate-buffered saline
PC: procyanidins
PKC: protein kinase C
RIPA: radio immunoprecipitation assay
ROS: reactive oxygen specie
SDS-PAGE: sodium dodecyl sulfate-polyacrylamide gel electrophoresis
TLR4: Toll-like receptor 4
TNF-α: tumor necrosis factor α
