# Supplementary material for: Procyanidins alleviates morphine tolerance by inhibiting activation of NLRP3 inflammasome in microglia
Source: J Neuroinflammation. 2016 Mar 1;13:53. doi: 10.1186/s12974-016-0520-z (PMC4774188; doi:10.1186/s12974-016-0520-z)
Supplement: Additional file 1: — Figures S1 and S2. BV2 cells were treated with rotenone for 6 h with or without the pre-treatment of procyanidins. We found that procyanidins could inhibit the upregulation of ROS which induced by rotenone. Additional controls with procyanidins treatment alone did not affect the baseline levels of NLRP3, pro-casp-1, casp-1, pro-IL-1β, IL-1β, and TNF-α. (DOCX 237 kb) [file 12974_2016_520_MOESM1_ESM.docx]

**Procyanidins alleviates morphine tolerance by inhibiting activation of** **NLRP3** **inflammasome in microglia**

Yang Cai^1†^, Hong Kong^1†^, Yin- Bing Pan^2†^, Lai Jiang^1^, Xiu-Xiu Pan^1^, Liang Hu^1^, Yan-Ning Qian^2^, Chun-Yi Jiang^1*^ and Wen-Tao Liu^1*^

^1^ Jiangsu Key Laboratory of Neurodegeneration, Department of Pharmacology, Nanjing Medical University, Nanjing 210029, China

^2^ Department of Anesthesiology, The First Affiliated Hospital of Nanjing Medical University, Nanjing 210029, China

^†^ Authors equally contribute to this work

^*^ Corresponding authors, addressed to: 140 Han-Zhong Road, Nanjing 210029, China

Email: jcy@njmu.edu.cn (C.J.); painresearch@njmu.edu.cn (W.L.)

Tel: +86-25-86862127; Fax: +86-25-86862127

**Supplementary Data**

**
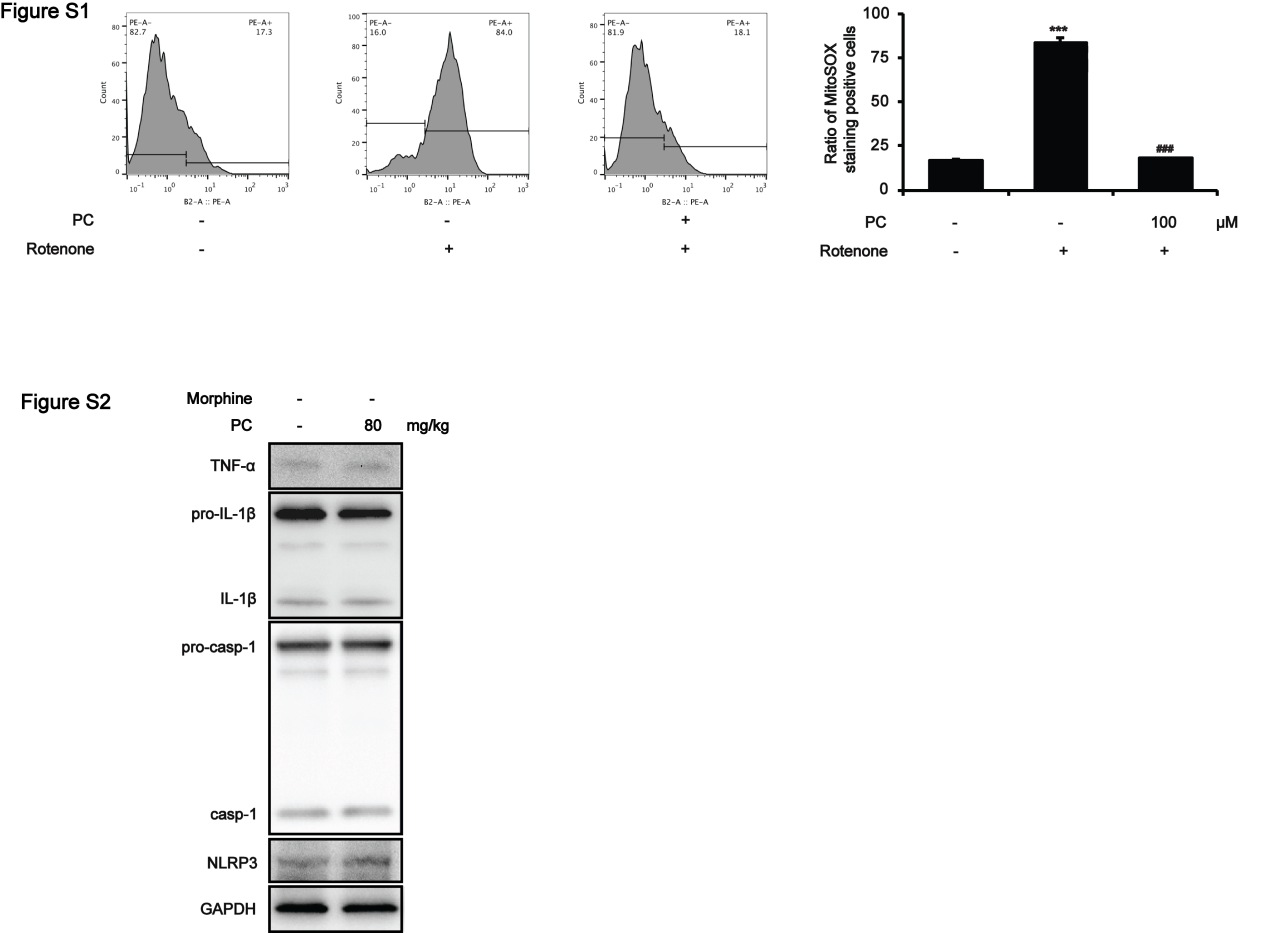
**

Figure S1. BV2 cells were treated with rotenone for 6h with or without the pre-treatment of procyanidins. We found that procyanidins could inhibit the up-regulation of ROS which induced by rotenone.


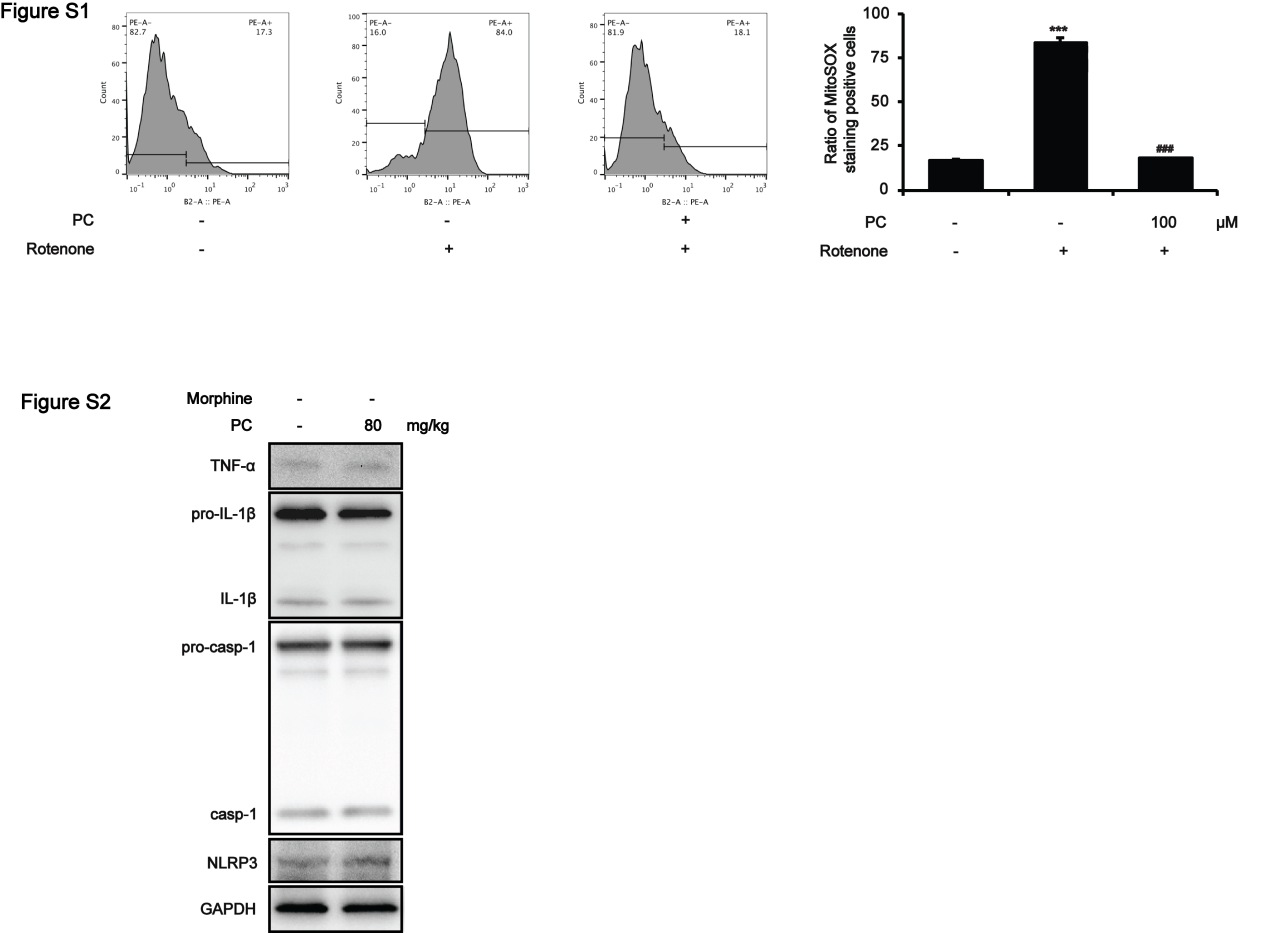


Figure S2. Additional controls with procyanidins treatment alone did not affect the baseline levels of NLRP3, pro-casp-1, casp-1, pro-IL-1β, IL-1β, and TNF-α.
